# A Community-Based Reproductive Health Care Model Effectively Enhances Reproductive Health Among Lahu Women in Northern Thailand

**DOI:** 10.1007/s40615-024-01959-5

**Published:** 2024-02-29

**Authors:** Soontaree Suratana, Waraporn Boonchiang, Tawatchai Apidechkul, Warangkana Naksen, Thanatchaporn Mulikaburt, Pimpisa Chomsri, Mullika Matrakul

**Affiliations:** 1 Faculty of Public Health, Chiang Mai University, 239 Huay Kaew Road, Muang District, Chiang Mai, 52000 Thailand; 2 School of Health Science, Mae Fah Luang University, Chiang Rai, 57100 Thailand; 3 Center of Excellence for Hill-Tribe Health Research, Mae Fah Luang University, Chiang Rai, 57100 Thailand; 4 School of Nursing, Mae Fah Luang University, Chiang Rai, 57100 Thailand

**Keywords:** Effectively, Community, Reproductive health, Model, Lahu women

## Abstract

**Background:**

Inadequate and delayed access to sexual and reproductive health services among the Hill Tribe population can be attributed to the intersection of socioeconomic challenges and distinct cultural practices. To address this limitation and create a tailored model capable of addressing it, this study assesses the effectiveness of the Community-Based Reproductive Health Care Model (CRHC) in enhancing reproductive health knowledge, attitudes, and practices among Lahu women, a prominent hill tribe population in Northern Thailand.

**Methods:**

Implementing the CRHC model includes training programs for community influencers and subsequent education for Lahu women using culturally adapted courses. The effectiveness of the model is assessed through pre-test and post-test comparisons of knowledge, attitudes, and practices related to reproductive health care and analyzed using paired *t*-tests and repeated ANOVA.

**Results:**

The scores for knowledge, attitudes, and practices among Lahu women changed from 8.92 ± 2.02, 52.99 ± 5.54, and 27.76 ± 6.67 to 10.47 ± 2.32 (*p* < 0.001), 56.61 ± 5.54 (*p* < 0.001), and 29.47 ± 6.76 (*p* = 0.030), respectively. Significant improvements are observed in these areas, particularly in maternal health practices among pregnant Lahu women (*n* = 11). This study additionally evaluates the model’s impact on the healthcare system by analyzing changes in government performance indexes, showing increased access to high-quality antenatal care and contraceptive usage. This study highlights the challenges faced by hill tribe populations in accessing healthcare, emphasizing the need for tailored reproductive health education and the importance of addressing health insurance barriers.

**Conclusion:**

The CRHC model’s success illustrates the potential of community-based, culturally sensitive interventions in improving reproductive health outcomes, providing valuable insights for similar interventions in other indigenous or marginalized communities.

## Introduction

Sexual and reproductive health is one of the essential elements of well-being, directly reflecting the quality of life of individuals and their families [1]. The United Nations (UN) recognizes this issue of sexual and reproductive health and has included it in the Sustainable Development Goals (SDGs), which member countries have agreed to improve as the targets are set [2, 3]. Reproductive health issues are the leading cause of illness and death among women of reproductive age worldwide [4]. Several studies indicate that lower socioeconomic status correlates with inadequate and delayed access to sexual and reproductive health services. This includes delayed detection of cervical cancer, limitations in preventing and treating reproductive system infections and sexually transmitted diseases (STIs), and reduced attention to gender-based violence. For example, around 90% of the four most common curable STIs were detected in populations residing in low- and middle-income countries. In several low- and middle-income nations, the prevalence of trichomoniasis ranged from 9 to 40%, in contrast to the lower rates of 1 to 4% observed in high-income countries. In addition, research has uncovered a negative correlation between socioeconomic status and adverse pregnancy outcomes, including stillbirth and low birth weight. A recent study has demonstrated that economic downturns in low- and middle-income countries can exacerbate under-5 mortality rates through their impact on dietary, environmental, and healthcare access factors. These mortality rates were observed to be 0.5% in high-income countries, in stark contrast to 6.81% in low- and middle-income countries [5]. Similarly, a significant portion of maternal and child mortality in Thailand can be attributed to inadequate access to sexual and reproductive health services.

Thailand has established a strategic plan to address this health issue, as outlined in its second National Reproductive Health Development Policy and Strategy (2017–2026). This plan aims primarily to enhance the approaching healthcare system, thereby broadening access to sexual and reproductive health services for all women in need [6]. The desired outcomes of this strategic plan include reducing unintended pregnancies among young women and improving preventable factors associated with maternal and child mortality for all women in the country [7]. However, the implementation has been successful in most of the Thai population, except for some ethnic groups who have distinct cultures, beliefs, and practices regarding sexual and reproductive health care [8–11]. For example, in the general Thai population, 75.78% of women completed five or more antenatal care (ANC) visits, whereas among women from hill tribes, the corresponding percentage was lower at 58.1%. Likewise, the prevalence of adolescent mothers, defined as those who became pregnant before the age of 20 years, was notably lower in the general Thai population at 2.87%, in contrast to a higher rate of 18.1% among women from hill tribes [12]. The latest report from 2019 indicates that approximately 3.5 million individuals from hill tribes live in Thailand, classified into six main groups: Akha, Lahu, Hmong, Yao, Karen, and Lisu [13]. Each tribe has unique culture, beliefs, and practices regarding sexual and reproductive health [14, 15]. Due to the presence of these distinct cultural elements, a significant portion of the targeted population, particularly Hill tribe adolescent women, continues to face challenges in accessing standard, free sexual, and reproductive health services [16].

Given the uniqueness of hill tribe cultures, beliefs, and practices concerning sexual and reproductive health, the Lahu people have been identified as having the poorest access to these services among the tribes [17]. The Lahu, the second-largest group among the hill tribes and the most socio-economically disadvantaged [18], is mainly concerned. For instance, a prior study conducted in Mae Suai District, where 67% of the population belongs to seven tribes—namely, Akha, Lahu, Karn, Lisu, Yao, Chinese-Yunnan, and Hmong—demonstrates this diversity. The work performance report on antenatal care and delivery services at Mae Suai District Hospital, predominantly serving Hill Tribe patients (over 75%), revealed a significant proportion of preterm deliveries and low birth weight cases, approximately 28–40 annually. Additionally, it was observed that only 40–61% of pregnant women completed five or more antenatal care visits, and there was a high incidence (18–23%) of pregnancies in women under the age of 20 [19]. These data suggest the presence of health inequalities that require attention and improvement in the existing reproductive healthcare, antenatal care, and delivery services offered by district hospitals.

Therefore, improving sexual and reproductive health within this population is a pressing issue among healthcare professionals. This study aimed to develop and evaluate the effectiveness of a Community-Based Reproductive Health Care Model (CRHC) in addressing these challenges by encouraging full involvement from all community stakeholders, especially community leaders, in the programs.

## Methods

### Study Design

The development and testing of the Community-Based Reproductive Health Care Model (CRHC) were conducted based on a participatory action research approach at the Lahu village named Khun Suai in the Wawee sub-district, which has 408 members. Given the significance of community leaders, religious figures, and village health volunteers in shaping and influencing the behavioral changes of community members, especially in matters related to health and cultural beliefs within the Lahu culture, these specific community members were selected to initiate the situation analysis. The situation analysis began with 27 community members consisting of ten Lahu women, five of their family members, and 12 community influencers, who are a community leader, a reverend, village health volunteers, a public health officer, nurses, a teacher, and a member of a nongovernmental organization (NGO).

This involved surveying the current sexual and reproductive health problems, factors contributing to these issues, and the type and characteristics of a healthcare model that could be successfully established within the community to solve or mitigate these problems. The Knowledge, Attitude, and Practice (KAP) tool was chosen as a practical framework for gathering information about the knowledge, viewpoints, beliefs, attitudes, and behavioral practices of Lahu women concerning challenges in sexual and reproductive health problems. A previous study elucidates the relationship between knowledge, attitude, and behavior [20]. The information gathering was used to initiate and develop this mode. The participants involved in the model development comprised healthcare staff (including a public health officer and nurses), significant community influencers, Lahu women, and their families.

A pre-test and post-test quasi-experimental design was employed to evaluate the effectiveness of implementing the CRHC model in four Lahu villages within the Wawee sub-district from May to November 2022.

### Setting and Samples

In Wawee sub-district, Mae Suai district, Chiang Rai Province, there are 25 villages with 17,439 people. Most of the hill tribe population in this sub-district are Akha and Lahu. Four Lahu villages—Thungprao, Khun Suai, Huaimasang, and Huaikhilek Mai—with populations of 624, 408, 1,069, and 722 members, respectively, were selected based on a purposive random sampling method and the criterion that the Lahu population was willing to participate in this model. Sample sizes were calculated based on the information obtained from the previous work [21] and using the EPI info program with the type I error of 0.05 and the power of 0.8. Therefore, a minimum of 36 women from each village should be selected using a simple random sampling technique. Given that puberty typically commences in Thai females at the age of 12 [22], while the phase of high-risk pregnancy predominantly affects women aged 35 and older [23], the study’s inclusion criteria encompassed Lahu women aged between 12 and 35 who possessed proficiency in Thai and were willing to commit to the study’s full 6-month duration.

### The Community-Based Reproductive Health Care Model (CRHC) Implementation

Based on the PAR approach, the CRHC model aimed to develop and consecutively enhance community-based initiatives to address knowledge gaps, improve attitudes, and promote appropriate practices related to reproductive health care that public health officials inadequately provide. It should be emphasized that in this community, the influencers of these villages, including community leaders, religious figures, and village health volunteers, are instrumental in driving behavioral change.

Training programs were implemented to empower these influencers, encompassing the “Train the Trainer” course and the “Mae Ying Roo Jing Reung Phet” course, the latter of which was developed based on the Applied international technical guidance on sexuality education [24]. The “Mae Ying Roo Jing Reung Phet” course comprises seven modules, which are as follows: (1) exploring “Reproductive Health” in Lahu Women; (2) understanding the “CRCH-Model”: A Role Model for the Community; (3) Changes as I Grow Up; (4) Adolescents’ Guide to Preventing Pregnancy; (5) AIDS and Sexually Transmitted Diseases: What You Need to Know; (6) Choosing the Right Birth Control: Preventing Pregnancy and Disease; and (7) Equipping Yourself for Professional Motherhood.

Participants who provided informed consent were enrolled in the “Mae Ying Roo Jing Reung Phet” course, facilitated by qualified trainers. This approach aimed to build upon existing knowledge, ensuring a gradual and comprehensive understanding.

Activities were strategically scheduled after Sunday Christian religious ceremonies or in venues arranged by community leaders, ensuring consistent and significant participant attendance. The success of these initiatives depended on influential community members leading the way in spreading essential information.

### Research Instruments

The data in this study were collected using a structured questionnaire divided into four sections and comprised 65 questions. This questionnaire was piloted within the community for clear, relevant, and reliable data. The first section aimed to collect sociodemographic and general information about the participants, consisting of 25 questions indicating age, marital status, pregnancy history, number of children, prevention of STDs, contraceptive use, occupation, education, income, and health insurance coverage. The second, third, and fourth sections encompass the assessment of participants’ reproductive healthcare knowledge, attitudes toward reproductive healthcare, and the frequency of reproductive healthcare practices, respectively. Scores obtained from the second section were categorized using Bloom’s taxonomy [25], while scores from the third and fourth sections were grouped and interpreted based on findings from a previous study [26].

The second section pertained to the reproductive healthcare knowledge of the selected women, comprising 15 closed-ended (yes/no) questions. These questions probed their understanding of STD prevention, condom use, contraceptive pills/injections, and knowledge about pregnancy and receiving antenatal care (ANC). The results were categorized into three levels based on the scores obtained: (i) low knowledge, indicating a need for knowledge improvement (score less than 9), (ii) moderate knowledge (score 9–11), and (iii) good knowledge (score 12–15).

The third section inquired about the participants’ attitudes toward reproductive healthcare. It consisted of 15 closed-ended questions on a rating scale ranging from 1 to 5, where 5 indicated strong agreement (disagreement), 3 indicated moderate agreement, and 1 indicated strong disagreement (agreement). The obtained data were categorized into three levels of attitudes: poor (15–35 points), moderate (36–55 points), and good (56–75 points).

The last section comprised ten closed-ended questions on a frequency scale ranging from 1 to 5, where 1 indicated “never done,” and 5 signified “regularly done.” The scores were categorized into three levels of practice: a score from 10 to 23.33 indicated that practices required improvement, 23.34 to 36.67 indicated fair practicing ability, and 36.68 to 50 indicated good practice. Additionally, five closed-ended frequency-based questions were employed concurrently to survey pregnant women about their maternal health habits. The results were divided into three tiers: scores ranging from 5.00 to 11.66 suggested a need for enhanced practice, scores from 11.67 to 18.33 denoted a moderate level of practice, and scores from 18.34 to 25.00 signified good practice.

Content validity was ensured through expert reviews from various fields, resulting in an index of congruence (IOC) of 0.87, indicating strong content validity. Reliability was confirmed by administering the questionnaire to 30 individuals with similar characteristics, revealing acceptable Cronbach’s alpha (*α* = 0.84) for attitude and practice items and a reliability coefficient of 0.71 for knowledge-related items. This establishes the questionnaire’s consistency and reliability for data collection.

### Data Analysis

Comparative analyses of reproductive healthcare knowledge, attitudes, and behaviors before, 1 month after, and 6 months after the trial were conducted using the paired *t*-test and repeated measures ANOVA statistics. Data analysis was conducted using SPSS 21.0 statistical software (IBM Inc., Chicago, USA), and statistical significance was determined at a significance level of *p* < 0.05. Means and standard deviations were reported for continuous data with a normal distribution, while medians and interquartile ranges (IQRs) were employed for continuous data with a skewed distribution.

## Results

**Fig. 1:**
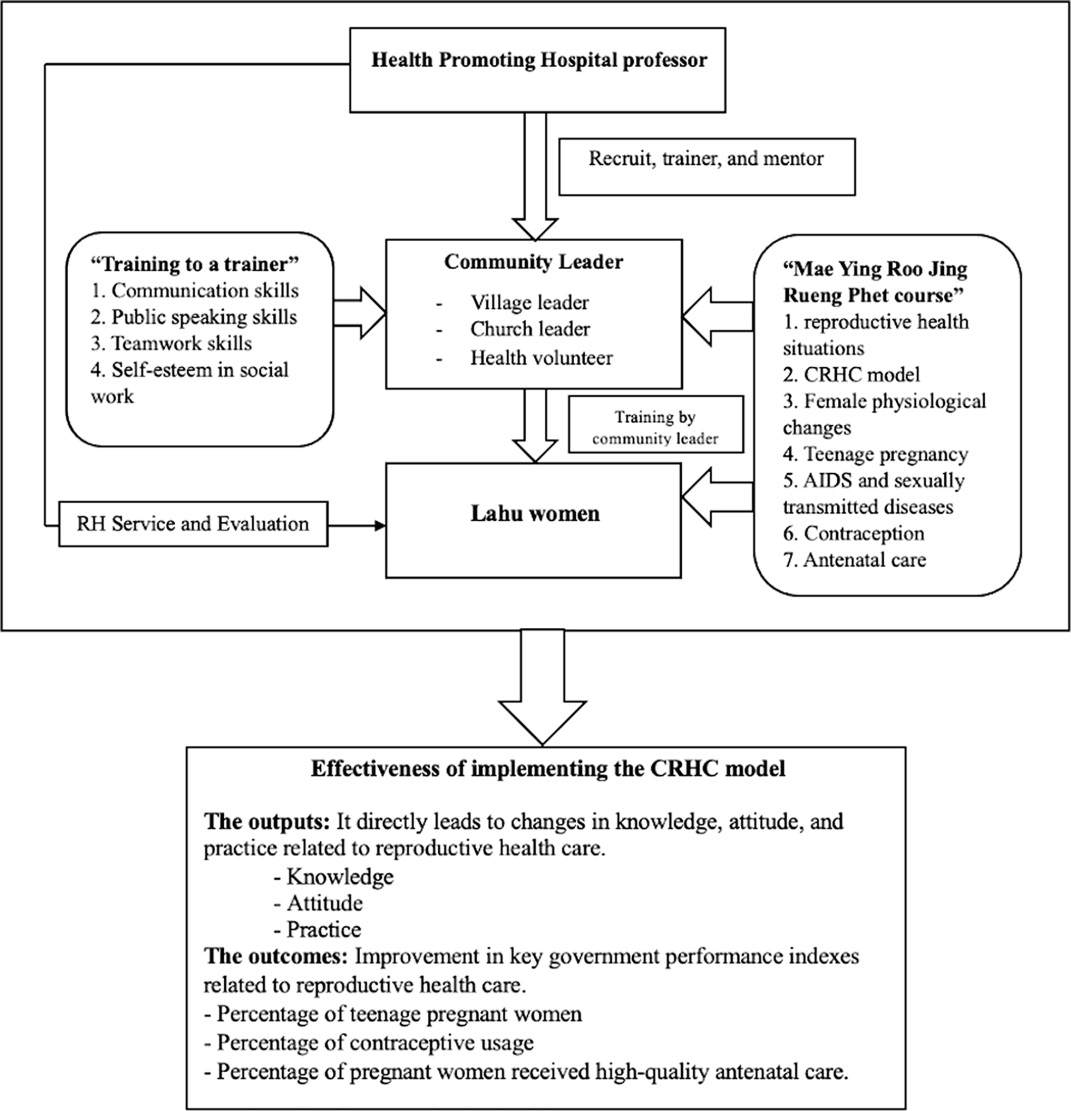
The working process of the CRHC (Community-Based Reproductive Health Care)

This study commences by implementing the CRHC model, as illustrated in Fig. 1. In each village, selected community influencers who serve as trainers actively participate in two courses: the “Train the Trainer” and “Mae Ying Roo Jing Reung Phet.” Subsequently, these trainers further educate the reproductive Lahu participants using the “Mae Ying Roo Jing Reung Phet” course. The effectiveness of the implemented model is demonstrated through the changes observed in the knowledge, attitude, and practice scores of the trained women, as well as the alterations in maternal health practice scores among pregnant women who underwent training. Additionally, this study examines the impact of the CRHC model implementation on the healthcare system by evaluating changes in government performance indexes, which are also reported in this work.

### The Sociodemographic Characteristics of Participants

The sociodemographic characteristics of all participants are detailed in Table 1. This study involved 146 reproductive Lahu women aged between 12 and 35, with an average age of 21.7. Among these participants, approximately half were teenagers, while the other half were adults. Around 40% of the reproductive women were single, and nearly 50% of them had no children. More than half of the participants reported being married and having 1–5 children. The majority of the reproductive Lahu women were students, while others worked as housewives or agriculturists or had no permanent job. About 90% of these women had completed primary or secondary school education. Notably, 58.9% of the reproductive Lahu women participating in this study did not have health insurance, while 37% had universal health insurance.

**Table 1:** Sociodemographic attributes of the Lahu women chosen to participate in this study

Characteristics	*n*	%
Total	146	100.0
Age (years) (mean = 21.7 S.D. = 7.2)		
12–20	72	49.3
21–35	74	51.7
Number of children		
None	72	49.3
1–2	53	36.3
3–5	21	14.4
Marital status		
Single	64	43.8
Married	78	53.4
Others	4	2.8
Occupation		
Housewife	23	15.8
Student	85	58.2
Agriculturist	56	38.4
Daily employed	9	6.1
Education		
No education	17	11.7
Primary school	44	30.1
Secondary school	85	58.2
Health insurance		
No	86	58.9
Universal health insurance	54	37.0
Private insurance	6	4.1

### Implementing the CRHC Model Significantly Improves the Scores for Knowledge, Attitudes, and Practices Related to Reproductive Health Care Among Lahu Women

Regarding changes in knowledge scores related to reproductive health care among Lahu women, our results showed that their knowledge was low before implementing the CRHC model (8.92 ± 2.02). We observed a significant improvement in their knowledge after 1 month and 6 months of model implementation, with *p*-values of 0.004 and less than 0.001, respectively. It should be highlighted that the knowledge score related to reproductive health care among Lahu women was changed to a moderate level. As anticipated, the attitude scores significantly changed from 52.99 ± 5.54 before the program intervention to 55.21 ± 7.03 (moderate level) and 56.61 ± 5.54 (good level) when measured 1 month and 6 months after the intervention, respectively. While there were no significant changes in the practicing scores related to protective behaviors against sexually transmitted diseases, safe sex, the use of contraceptive agents, and the recording of menstrual cycles observed one month after participating in the model, these scores among the Lahu women significantly improved six months after the implementation of the CRHC model (Table 2).

**Table 2:** The impact of implementing the CRHC model on changes in knowledge, attitudes, and practices regarding reproductive health care among Lahu women (*n* = 146)

	Knowledge		Attitude		Practice	
	Mean (SD)	MD (95%CI) p-value	Mean (SD)	MD (95%CI) p-value	Mean (SD)	MD (95%CI)p-value
Before	8.92 (2.02)		52.99 (5.54)		27.76 (6.67)	
After 1st month	9.69 (2.41)	0.77 (0.19,1.34)^0.004^	55.21 (7.03)	2.22 (0.30, 4.14)^0.018^	27.81 6.67)	0.05 (− 1.49, 1.59)^0.951^
After 6th month	10.47 (2.32)	1.54 (0.94, 2.15) < ^0.001^	56.61 (5.54)	3.62 (2.21, 5.22)^<0.001^	29.47 (6.76)	1.71 (0.17, 3.25)^0.030^
1st vs. 6th month		0.77 (0.38, 1.17)^<0.001^		1.40 (− 0.34, 3.14)^0.160^		1.66 (0.12, 3.24) ^0.034^

### The Implementation of the CRHC Model Leads to a Significant Enhancement in Behavior Scores Concerning Maternal Health Practices in Pregnant Lahu Women

Among the reproductive women participating in the CRHC model, 11 were pregnant women. Their behaviors related to maternal health practices were also assessed and are presented in Table 3. Interestingly, significant improvements were observed in the scores for maternal health practices, which reflect their frequency of visiting antenatal care (ANC) services and following the recommendations outlined in the pregnancy guidebooks provided by hospitals and healthcare officers. These scores improved from 16.27 ± 4.31, defined as fair practice scores, to good practice scores of 20.81 ± 5.75 and 23.18 ± 1.25 when measured 1 month and 6 months after participating in this course, respectively. The analysis of repeated measures ANOVA revealed the practice scores of pregnant women. The Greenhouse–Geisser correction for within-subject testing was used due to a violation of sphericity. There was a statistically significant difference among the three-time points regarding pregnancy practice (*p* < 0.05) (Table 4). The average of the practice scores of pregnant women improved after the intervention.

**Table 3:** The impact of implementing the CRHC model on changes in maternal health practice scores among pregnant Lahu women (*n* = 11)

	Practice scores		*p*-value
	Mean (SD)	MD (95%CI; lower, upper)	
Before	16.27 (4.31)		
After 1st month	20.36 (2.16)	4.09 (1.58,6.60)	0.002
After 6th month	23.18 (1.25)	6.91 (4.40, 9.41)	< 0.001
6th vs. 1st month		2.82 (0.31, 5.32)	0.029

**Table 4:** Repeated measures ANOVA examining changes in knowledge, attitudes, and practices regarding reproductive health care scores among Lahu women at three-time points

Sources	Sum of squares (SS)	df	Mean square (MS)	*F*	*p*-value	Partial eta squared
*The knowledge scores of Lahu women (n* = *146)*						
Within group	173.374	1.659	104.487	24.567	< 0.001*	0.145
Error	1023.292	290	3.529			
*The attitude scores of Lahu women (n* = *146)*						
Within group	971.178	1.916	507.001	12.611	< 0.001*	0.080
Error	11,166.822	277.753	40.204			
*The practice scores of Lahu women (n* = *146)*						
Within group	277.621	2	138.811	3.328	0.037*	0.022
Error	12,095.712	290	41.709			
*The practice scores of pregnant women (n* = *11)*						
Within group	265.515	1.286	206.526	23.058	< 0.001*	0.698
Error	115.152	12.856	8.957			

^*^Statistical significance at 0.05

### Implementing the CRHC Model Resulted in Changes in Government Performance Indexes

Eight months after the implementation of the model, secondary data related to reproductive healthcare, as outlined in the government performance indexes, were collected and compared with data obtained four years before the introduction of this model into the healthcare system. These indexes include the frequency of accessing ANC services, the number of teenage pregnancies, and the percentage of contraceptive usage among reproductive women, reflecting their access to family planning (FP) services. The percentage of pregnant women receiving high-quality ANC services increased from 48.5 to 55.6% from 2018 to 2021, reaching 66.7% in 2022. However, only a slight reduction in teenage pregnancies was observed. In 2018, there were 13 pregnant teenagers out of a total of 46 pregnant women. In the subsequent years, from 2019 to 2022, there were 8 to 9 teenage pregnancies reported out of 30 to 45 pregnant women. The percentage of contraceptive usage among reproductive women also increased from 25.6 to 35.2% between 2018 and 2021, and it reached 46.0% in 2022 (Table 5).

**Table 5:** The changes in government performance indexes, including percentages of access to antenatal care (ANC), teenage pregnancy, and family planning (FP) services, were assessed before (in 2018, 2019, 2020, and 2021) and after (in 2022) the implementation of the CRHC model

Years	Total pregnant women	No. of pregnant women receiving high-quality ANC (%)	No. of teenage pregnancy (%)	Family planning	
				Total reproductive women	Contraceptive users (%)
2018	46	24 (52.2)	13 (28.3)	956	245 (25.6)
2019	45	25 (55.6)	9 (20.0)	973	304 (31.2)
2020	30	16 (53.3)	9 (30.0)	957	337 (35.2)
2021	33	16 (48.5)	8 (24.2)	920	318 (35.2)
2022	39	26 (66.7)	8 (20.5)	958	441 (46.0)

## Discussion

This study was conducted in Mae Suai District, Chiang Rai, where 18.94% of the ethnic population in Chiang Rai resides. A prior study [27] highlighted the issue of pregnant adolescents in this district, with 47.4% of pregnant women under the age of 20 being Lahu women. Cultural beliefs, particularly among the Lahu people, present challenges to accessing healthcare. Lahu women, who are part of the second-largest hill tribe in Thailand, often marry at a young age, the lack knowledge of family care, and face increased child mortality within the first year. Thus, there is a pressing need to educate and empower young people regarding reproductive health to prevent diseases, unwanted pregnancies, and unsafe abortions [28].

This study effectively introduced and implemented the Community Reproductive Health Care (CRHC) model, utilizing influencers as trainers for reproductive Lahu women. The model demonstrated significant improvements in knowledge, attitudes, and practices related to reproductive health care, particularly in maternal health practices for pregnant women. The positive impact is also reflected in government performance indexes, indicating increased access to antenatal care services and higher contraceptive usage among reproductive women. These findings underscore the CRHC model’s effectiveness in promoting reproductive health outcomes and suggest its potential for more comprehensive community application.

It is well-established that hill tribe populations face restricted access to healthcare services, resulting in a higher likelihood of inappropriate treatment [29, 30]. Moreover, geographical isolation, poverty, culture, and beliefs pose significant challenges for hill tribe populations in achieving optimal health, including healthcare services for maternal and childhood [31]. The demographic data of the study participants, predominantly reproductive-age women between 12 and 35 years, highlights an essential period for reproductive health education. This observation is consistent with previous research on various racial and ethnic populations in the United States, which found that the primary reproductive age span was between 25 and 34 years [32]. Studies from both the US and the UK demonstrate that knowledge, awareness, and perceptions of reproductive health among women of reproductive age vary across racial and ethnic groups [33–35]. Consequently, there is a need for reproductive health education specifically tailored to the community’s diverse life stages and experiences [36]. This is especially critical for the hill tribe populations in Northern Thailand, who have not had a specialized healthcare model addressing the needs of reproductive women until now.

Our study emphasizes the health insurance challenges within ethnic groups, particularly among hill tribe populations. The absence of a Thai ID card results in the inability to access healthcare services fully, including those related to maternal and child healthcare. The Thai government has recognized this issue and introduced a universal healthcare coverage scheme (UC) for individuals possessing a Thai pink ID card, the identity document issued to both stateless and alien populations. However, approximately 30% of the hill tribe population lacks this card [13]. Therefore, it is imperative to encourage them to purchase low-cost non-Thai resident health insurance to ensure they have proper access to healthcare services. This problem is similar to healthcare challenges encountered by ethnic, internal, and external migrants and refugees in various countries, including the US, European nations, and China [33, 34, 37]. These findings highlight the link between issues of citizenship and ethnicity. Respondents reported experiencing stigma, discrimination, and a lack of social support. A deeper analysis of stateless ethnic groups could accelerate efforts to resolve long-standing citizenship issues and reduce ethnic disparities [38, 39]. Therefore, it is essential to raise awareness among ethnic groups about the importance of health insurance and conduct health promotion activities in a context appropriate for the community.

The post-implementation improvements in knowledge and attitudes towards reproductive health with the CRHC model are promising. A notable achievement is the significant improvement in behavior scores regarding maternal health practices among pregnant Lahu women, particularly in increased antenatal care visits and adherence to pregnancy guidebooks. This underlines the value of targeted interventions for pregnant women. Similarly, the Participatory Education on Adolescent Reproductive Life (PEARL) model used with adolescent migrants in Thailand showed enhanced knowledge, attitudes, and behavior in preventing unintended pregnancies [40]. Various country-specific interventions have successfully improved knowledge and attitudes toward reproductive health care. For instance, a study on domestic migrants in China using a comprehensive sexual and reproductive health/family planning intervention markedly improved participants’ knowledge and attitudes about sexual reproductive health and contraception use [41]. In India, the PRACHAR model led to increased knowledge and attitudes and a focus on behavior change among young married couples [42]. Moreover, a school-based comprehensive sexuality education program in rural Uganda improved adolescents’ understanding of SRH and awareness of unwanted pregnancies and sexually transmitted infections [43]. This study examines health insurance challenges within ethnic groups, specifically on hill tribe populations, and draws parallels with migrants and refugees in various countries. The findings reveal a correlation between their increasing knowledge and attitudes and improved reproductive health knowledge and behaviors. Post-implementation assessments of the CRHC model demonstrate promising enhancements in reproductive health knowledge and behaviors, highlighting the significance of targeted interventions, such as increased antenatal care visits, in promoting maternal and child health.

Insufficient antenatal care (ANC) visits have been linked to adverse health outcomes, such as increased risks of low birth weight, preterm delivery, abortion, and perinatal mortality. The post-implementation changes in government performance indexes reflect the broader systemic impact of the CRHC model, evident in the increased percentage of pregnant women receiving high-quality antenatal care and using contraceptives. High-quality antenatal care is crucial for the health and well-being of both mother and child, forming the foundation of maternal and child health programs globally. A study from Ghana reported that poor and average-quality ANCs were significantly associated with higher chances of developing pregnancy-related complications [44]. Previous studies from various low- and middle-income countries have consistently shown a reduction in maternal and neonatal mortality, morbidity, and adverse events among those with adequate antenatal care visits. Enhancing access to quality prenatal care and addressing teenage pregnancy are priorities, especially in developing countries [45, 46].

These findings have profound implications for health policy, particularly in designing and implementing community-based health interventions. The CRHC model’s success in improving reproductive health outcomes among Lahu women can serve as a template for similar interventions in other indigenous or marginalized communities. The potential presence of confounders, particularly the fact that some participants are students, may introduce interference due to their exposure to the content covered in school courses. Although the results are encouraging, the study’s focus on a specific demographic group within a single community limits its generalizability. Future research should investigate the adaptability and effectiveness of the CRHC model in different settings. Moreover, the slight decrease in teenage pregnancies indicates a need for more targeted interventions in this area.

## Conclusion

Implementing the CRHC model in the Lahu community highlights the potential of community-based, culturally sensitive interventions to improve reproductive health outcomes. Its success provides valuable insights for health practitioners and policymakers in similar contexts. It is important to acknowledge that several factors could contribute to variations in the government performance indexes, including variables such as the annual number of total reproductive women and total pregnant women, which can influence both the percentages of contraceptive usage among reproductive women and the incidence of teenage pregnancies. Consequently, the need for an effective measurement procedure that accurately reflects the impact of this model implementation, in addition to the government performance indexes, necessitates further investigation.

## Data Availability

Due to ethical concerns with participants’ data and privacy, the datasets obtained and/or analyzed during the current study are not publicly available. The information and materials discussed in the manuscript are, nevertheless, accessible upon the relevant author’s justifiable request to the corresponding author, Associate Professor Dr. Waraporn Boonchiang (E-mail: Waraporn.b@cmu.ac.th).
